# The molecular evolutionary dynamics of the vomeronasal receptor (class 1) genes in primates: a gene family on the verge of a functional breakdown

**DOI:** 10.3389/fnana.2014.00153

**Published:** 2014-12-12

**Authors:** Anne D. Yoder, Peter A. Larsen

**Affiliations:** Department of Biology, Duke UniversityDurham, NC, USA

**Keywords:** olfaction, gene family evolution, positive selection, adaptation, lemurs, draft genomes

## Abstract

Olfaction plays a critical role in both survival of the individual and in the propagation of species. Studies from across the mammalian clade have found a remarkable correlation between organismal lifestyle and molecular evolutionary properties of receptor genes in both the main olfactory system (MOS) and the vomeronasal system (VNS). When a large proportion of intact (and putatively functional) copies is observed, the inference is made that a particular mode of chemoreception is critical for an organism’s fit to its environment and is thus under strong positive selection. Conversely, when the receptors in question show a disproportionately large number of pseudogene copies, this contraction is interpreted as evidence of relaxed selection potentially leading to gene family extinction. Notably, it appears that a risk factor for gene family extinction is a high rate of nonsynonymous substitution. A survey of intact vs. pseudogene copies among primate vomeronasal receptor Class one genes (V1Rs) appears to substantiate this hypothesis. Molecular evolutionary complexities in the V1R gene family combine rapid rates of gene duplication, gene conversion, lineage-specific expansions, deletions, and/or pseudogenization. An intricate mix of phylogenetic footprints and current adaptive landscapes have left their mark on primate V1Rs suggesting that the primate clade offers an ideal model system for exploring the molecular evolutionary and functional properties of the VNS of mammals. Primate V1Rs tell a story of ancestral function and divergent selection as species have moved into ever diversifying adaptive regimes. The sensitivity to functional collapse in these genes, consequent to their precariously high rates of nonsynonymous substitution, confer a remarkable capacity to reveal the lifestyles of the genomes that they presently occupy as well as those of their ancestors.

## Introduction

Vomeronasal receptor (Class 1) genes (V1Rs) are one of five independently derived functional classes of chemoreceptor genes in the olfactory system of mammals. Together with vomeronasal receptor (Class 2) genes (V2Rs) and a small family of formyl peptide receptors (FPRs), V1Rs are found clustered within an anatomical structure referred to as the vomeronasal organ (VNO). The two additional classes of receptor, the odorant receptors (ORs) and trace amine-associated receptors (TAARs) are found in the nasal epithelium. All five are G-protein coupled receptors (GPCRs), and as such, have structural features that are shared with other chemosensory genes such as sweet and bitter taste receptors. All GPCRs are structured to detect an enormous variety of extracellular signals, including photons, ions, small organic molecules and entire proteins. The structure of a GPCR can be divided into three parts: the extracellular region, consisting of the N terminus and three extracellular loops, the transmembrane region (TM), consisting of seven α-helices; and the intracellular region, consisting of three intracellular loops and the C-terminal tail. ORs fall into Class A GPCRs wherein the TM regions comprise a consensus “ligand-binding cradle” that constitutes the bottom of the ligand-binding pocket within the TM bundle. The third TM helix (TM3) has a central role as a structural and functional hub (Venkatakrishnan et al., 2013), putatively placing it under very strong purifying selection given the essential nature of its structure and placement to the stability of the ligand-binding cradle (e.g., Herberger and Loretz, 2013). These insights into the structure-function relationships of the GPCRs have thus yielded new perspectives for molecular evolutionary investigations of the types and levels of selective forces as they act on V1R and other chemosensory genes within mammals generally, and in this review, primates specifically.

The olfactory system is divided into two general systems, the main olfactory system (MOS) and the vomeronasal system (VNS), which though derived from non-homologous evolutionary pathways, are intimately related both ontogenetically, sharing similar molecular and cellular processes, and evolutionarily, showing similar adaptations to changing ecological scenarios. This system has been referred to as “dual olfaction” (Suárez et al., 2012) and thus lessons learned from one can reasonably be applied to the other. This view therefore differs from the so-called “differential tuning” hypothesis proposed by Grus and Zhang (2008) in which the authors make note of the fact that though functioning within a single morphological and behavioral system, the MOS and VNS are putatively under different selective regimes with the MOS tuned to environmental and volatile odorants and the VNS to non-volatile semiochemicals that govern many conspecific (e.g, mating) and heterospecific (e.g., predation) interactions. Evidence continues to accumulate that the VNS is finely tuned to social cues and thus under strong lineage-specific selection (e.g., Garrett and Steiper, 2014) and is changing more rapidly than the more conserved ORs (D’Oliveira Albanus et al., 2014). It has been further demonstrated that within the VNS, the two receptor types, V1Rs and V2Rs, derive from different evolutionary precursors with each receptive to different ligand types: V1Rs tuned to detect small chemicals and V2Rs to detect proteinaceous ligands (Giorgi et al., 2000; Leinders-Zufall et al., 2000, 2009; Pantages and Dulac, 2000; Rodriguez et al., 2000; Kouros-Mehr et al., 2001; Del Punta et al., 2002; Giorgi and Rouquier, 2002; Grus and Zhang, 2004; Grus et al., 2005, 2007; Nodari et al., 2008; Korsching, 2009; Haga et al., 2010; Isogai et al., 2011; Park et al., 2011; Wynn et al., 2012; Hohenbrink et al., 2012a, 2014; Haga-Yamanaka et al., 2014).

Though it has been proposed that a move from an aquatic to terrestrial lifestyle has driven an apparent shift from a predominantly V2R to V1R system in mammals, recent investigation of VR gene expression in snakes and lizards has overturned this hypothesis (Brykczynska et al., 2013). This study found that snakes and lizards retain an extremely limited number of V1R genes but exhibit a large number of V2R genes, including multiple lineages of reptile-specific and snake-specific expansions. Moreover, elegant *in vivo* work by Isogai et al. (2011) has shown that both receptor families are highly active in mice (Chamero et al., 2011). Collectively, the olfactory system allows for the detection of a multitude of odorants and pheromones that are environmentally transmitted and must be internally processed to identify everything from appropriate food sources to potential mates to predators. In short, this sensory system is critical for the survival of the individual and for the propagation of the species.

## “The dark matter of social neuroscience”

In a review of the role of oxytocin and vasopression in modulating social interactions and affilicative behavior among mammals, Insel (2010) adopted the term “dark matter” of social neuroscience to describe the “complex territory between perception and action” that governs social cognition. This conceptual framework also applies broadly to the olfactory system (Chamero et al., 2012). Olfaction is critical to an organism’s ability to survive, though in primates, there are multiple clues from anatomy, behavior, and the genome to suggest that the sensitivity of the system has diminished over their evolutionary history (Heymann, 2006; Dong et al., 2009), especially within the anthropoid primates. This decay of the olfactory system in catarrhine primates specifically was initially thought to be linked to the acquisition of trichromatic vision in old world monkeys and apes. In large part, this conclusion was supported by the observation that the howler monkey, the only New World monkey to possess trichromatic vision, showed a much higher proportion of OR pseudogenes than other NW monkeys (Gilad et al., 2004). More complete characterization of the ratio of intact to pseudogene copies and their distributions in both OW and NW anthropoids has overturned this conclusion, however (Gilad et al., 2007; Matsui et al., 2010).

In mammals more generally, numerous studies have found a remarkable connection between organismal lifestyle and molecular evolutionary patterns among olfactory receptors within the MOS and the VNS (Chamero et al., 2012; Ibarra-Soria et al., 2014; Sánchez-Andrade and Logan, 2014). In the case of the VNS, the fit between organismal lifestyle and genomic repertoire is interpreted to relate to pheromonal communication between “emitter” and “receiver”. Sex pheromones in particular play fundamental roles in mate choice in a system that is conserved across the animal kingdom (Gomez-Diaz and Benton, 2013; Petrulis, 2013a,b; Liberles, 2014). Recent studies in the mouse model have shown that both the VNO and MOS are key to mediating social behavior (Mandiyan et al., 2005; Chamero et al., 2012) with *in vivo* studies of specific receptor function revealing an extraordinary specificity of receptor response to specific stimuli (Isogai et al., 2011). The latter study in particular was able to characterize the stimulus-response profiles in 88 VNO receptors, finding a mix of conspecific and heterospecific associations. The results illustrate a fascinating combination of specificity and generality. Whereas some receptors were exquisitely tuned to stimuli from conspecifics (e.g., certain receptors responded only to stimuli from conspecific females) or to heterospecific cues (e.g., some showed a unique association with distinct classes of predators such as snakes, but not others, such as fox), others were much more generalized. Notably, Isogai et al. (2011) found fundamental functional differences between the two classes of VRs, with V1Rs being the more generalized and V2Rs the more specialized. Due to their ability to distinguish among distinct structural classes of steroids, the authors surmised that V1Rs are uniquely geared to detect the physiological status of an individual, whether conspecific or heterospecific. Taken as a whole, the increasing sophistication of studies focused on VNO function reveals a field poised for an explosive era of discovery by uniting and reciprocally illuminating the fields of neuroendrochrinology, neuroimmunology, neuroethology, and social behavior. This integrative approach holds tremendous promise for unlocking the secrets of what Insel (2010) has called “the dark matter of social neuroscience” (Chamero et al., 2012).

## A molecular-evolutionary locomotive

In a landmark study of the V1R genes in mammals, Young et al. (2010) found remarkable patterns of both inter- and intra-specific variability among mammalian genomes, as mined from draft genomes available at that time. They discovered patterns wherein species-specific subfamilies and “semi-private” alleles are common among the 37 mammals sampled with approximately 80% of V1R clades being species-specific. These molecular evolutionary complexities in the V1R gene family have been repeatedly noted and hypothesized to differentially combine rapid rates of gene duplication, gene conversion, lineage-specific expansions, deletions, and/or pseudogenization (e.g., Rodriguez et al., 2002; Shi et al., 2005; Wynn et al., 2012; Brykczynska et al., 2013). This behavior is in keeping with patterns of gene family evolution in general. Demuth et al. (2006) has described the genomic “revolving door” of gene loss and gain that seems to characterize much of the human genome, as well as mammalian outgroups. In their comparison of human, chimp, mouse, rat, and dog, these authors found shifting patterns of expansions and contractions within the nearly 10,000 gene families compared by the study. Regarding primate evolution specifically, their study indicates that the human genome contains at least 1418 genes (6.4% of all known genes at that time) that do not have orthologs in the chimpanzee, with an even greater discrepancy found between mouse and rat. The authors raise the interesting point that duplication and losses within gene families have played a greater role than nucleotide substitution in generating evolutionary change across mammalian genomes, and in the human genome specifically.

The question thus arises: what are the forces that drive this molecular evolutionary locomotive? An obvious dichotomy to be considered is between neutral and adaptive processes (e.g., Demuth et al., 2006; Park et al., 2011; Wynn et al., 2012). The phylogenetic distribution within and among mammals for both ORs and VRs suggests a strong role for adaptation, both as individual gene classes (e.g., Shi et al., 2005; Verrelli et al., 2008; Luca et al., 2010; Matsui et al., 2010; Wang et al., 2010a,b; Yoder et al., 2014) and in concert with one another (Suárez et al., 2012). These patterns extend broadly across the animal kingdom. In a study that surveyed chemosensory signals and their receptors in the olfactory system of mice, insects, and nematodes, Ihara et al. (2013) review the current understanding of signaling molecules where the information stream from receptor to specific behavior has been characterized. They find an overarching pattern wherein each organism has evolved a chemosensory system that ideally suits its lifestyle or environmental conditions. Given their focus on model organisms, these authors were able to examine the precise molecular mechanisms that underlie the linkage from signaling mechanisms to behaviors. Another study of *in vitro* responses in the ORs of mouse and human found a high fidelity of OR orthologs in primates and rodents, demonstrating that amino acid changes can have dramatic functional consequences that are a reflection of niche and other species-specific demands (Adipietro et al., 2012). For non-model organisms, however, the correspondence among environment, genome, and behavior must be approximate and correlative.

In most cases, conclusions about relative adaptive fit of OR and VR repertoires have been drawn from the molecular characterization of intact vs. pseudogene copies within receptor classes (e.g., Lane et al., 2002; Rodriguez et al., 2002; Pfister and Rodriguez, 2005; Shi et al., 2005; Young et al., 2005; Liman, 2006; Niimura and Nei, 2006; Grus et al., 2007; Young and Trask, 2007; Olender et al., 2008, 2012; Johnstone et al., 2009; Nikaido et al., 2013). Specifically, when there is a large proportion of intact vs. pseudogene copies, the inference is made that a particular mode of chemoreception is critically linked to the organism’s lifestyle and is thus under strong positive selection. Conversely, when the receptors in question are found to have a disproportionately large number of pseudogene copies, investigators interpret this as evidence of relaxed selection and concomitant drift in nucleotide substitutions. As a classic example of such inference, Gilad et al. (2004) interpreted the deterioration of the OR repertoire in Old World monkeys and apes (including humans) to have a functional correspondence with the acquisition of trichromatic vision in these but not other primates. Similarly, Zhao et al. (2011) interpreted the deterioration of the VNS in bats to be related to the acquisition of their novel communication and foraging system (although based on a limited sample). Numerous studies have noted a connection between nocturnality and a high proportion of intact VR genes (e.g., Wang et al., 2010a,b) based upon the assumption that nocturnal habits will place strong selective pressures on the olfactory system in lieu of the visual system.

Expansion and contraction in gene family evolution is likely universal in eukaryotes. In a detailed study of gene family histories in yeast, Ames et al. (2014) observed differential rates of both phenomena among and between gene families and species, finding that both appear to be correlated with species-specific adaptations. Moreover, this study found that gene families with highly specific functions (as would be the case with chemosensation) repeatedly and independently expanded in multiple species, thus suggesting common underlying adaptive pressures. Within the human lineage, at least 2.7% of the genome has been uniquely duplicated since the human-chimp divergence, undoubtedly reflecting many of the unique adaptations of the human lifestyle. Similarly, gene loss has also been proposed as an adaptive response to changing environmental conditions or adaptive strategies (Olson, 1999) though contraction has been more typically assumed to reflect a relaxation of selective pressure (e.g., Liman and Innan, 2003; Mundy and Cook, 2003; Mundy, 2006; Zhao et al., 2011).

The effects of positive selection are more easily conceived for expansion via gene duplication with subsequent amino acid changes to the newly duplicated gene. As an example, positive selection may be exerted on regions of a receptor gene where that receptor manifests its ligand-binding functions. If the sequence change expands the repertoire of chemosensory signals that can be detected, it follows that this will be advantageous to the organism and these changes will be fixed by positive selection. One possible example of this phenomenon can be inferred from a recent empirical study of the V1R gene family in strepsirrhine primates (the lemurs and lorises). In that study, the authors discovered a remarkable expansion of V1R genes in the strepsirrhines relative to other primates, finding a gene subfamily putatively unique to the strepsirrhine clade and designated as V1R*strep* (Yoder et al., 2014). Moreover, phylogenetic analysis of V1R*strep* revealed that a relatively ancient (>40 mya) gene duplication occurred within the lemur clade, resulting in two V1R*strep* subclades referred to as α and β. Presumably, this pattern of initial expansion followed by gene duplication and further expansion has conferred a functional advantage to the lemurs, a finding that is congruent with experimental work that has shown that orthologs are far more likely to correspond to a common odor than will paralogs within the same gene family (Adipietro et al., 2012).

## V1Rs within the primates: on the verge of a functional breakdown

**Figure 1:**
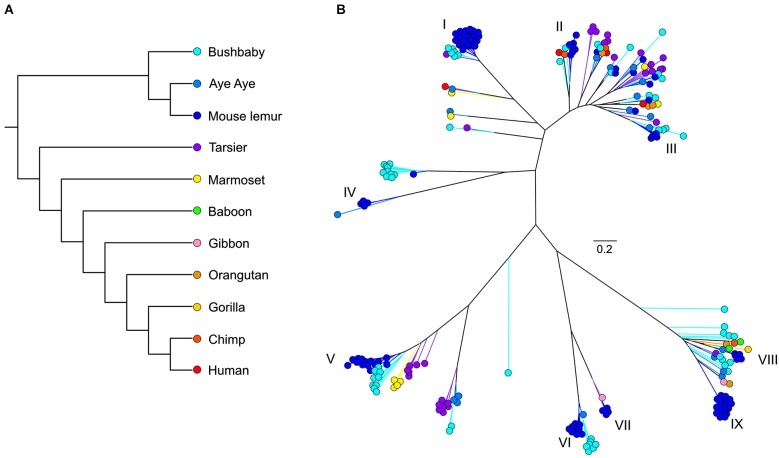
**(A)** Phylogeny of primate species discussed herein. **(B)** Unrooted maximum likelihood tree generated from RAxML analyses of primate V1R amino acid sequences. Roman numerals identify mouse lemur V1R subfamilies described in Hohenbrink et al. (2012b).

As with most mammals, the phylogenetic relationships among primate V1Rs do not closely follow those of the species that carry them. In other words, there is a significant mismatch between the V1R gene tree and the primate species tree. Both Table 1 and Figure 1 illustrate the phylogenetic patterns among all primate V1Rs mined from (predominantly) draft genomes available in Ensembl as of 2010 (Young et al., 2010). The figure on the left (A) illustrates the accepted primate species phylogeny whereas that on the right (B) illustrates the primate V1R gene tree. Figure 1 establishes both that the ancestral primate genome carried at least six subfamilies of V1R alleles, and that the strepsirrhines (illustrated in shades of blue) have a substantially higher number of copies than do the haplorrhines (all other colors). The figure thus illustrates differential patterns of expansion and contraction in the V1R system during primate evolutionary history.

**Table 1:** **Number of total and intact V1Rs identified by Young et al. (2010) in primate species**.

Species	Total V1Rs*	Intact V1Rs
Macaque	60	0
Gibbon	110	2
Gorilla	115	3
Human	116	3
Baboon	94	3
Chimpanzee	106	4
Orangutan	178	5
Marmoset	63	8
Tarsier	266	42
Bushbaby	133	78
Mouse lemur	259	214

* Values are median estimates (Young et al., 2010).

Changes in gene family copy number among species can evolve at rapid rates (e.g., Cutler et al., 2007; She et al., 2008). Indeed, comparisons among inbred strains of laboratory mice have revealed similar patterns within the V1R complex, further supporting the idea that rates of change can be very high for chemosensory genes (Wynn et al., 2012). It is noteworthy that one of the common “symptoms” of gene family extinction is a high rate of nonsynonymous substitution (Nielsen et al., 2005). As a specific case, Wynn et al. (2012) observed that 57% of total single-nucleotide polymorphisms (SNPs) in the VRs of mice are nonsynonymous substitutions for both wild populations and for inbred laboratory strains. The relationship between nonsynonymous and synonymous substitution rates has long been used as a measure for estimating the strength and directionality of selection (i.e., purifying vs. positive). Referred to as the dN/dS rate ratio, this measure has served as a reliable (though approximate) method in studies across virtually all of metazoan life (Yang, 1998; Vamathevan et al., 2008; Yang et al., 2009; Yang and dos Reis, 2011) and is applicable to primate VRs (Shi et al., 2005; Hohenbrink et al., 2012b; Yoder et al., 2014). One consequence of this positive selection engine is that the pairwise genetic distances among amino acids can be greater than their underlying nucleotide distances (e.g., see Table 1 in Yoder et al. (2014); whereas pairwise nucleotide distances tend to range up to a maximum of 13% in that study, those for amino acids can exceed 26%). It therefore stands to reason that these high rates of amino acid change must place VRs on the verge of functional collapse. Even the slightest relaxation of selective pressure can tip the balance towards pseudogenization and gene family contraction. In the words of Liman (2006, 2012) it is a “use it or lose it” system.

**Figure 2:**
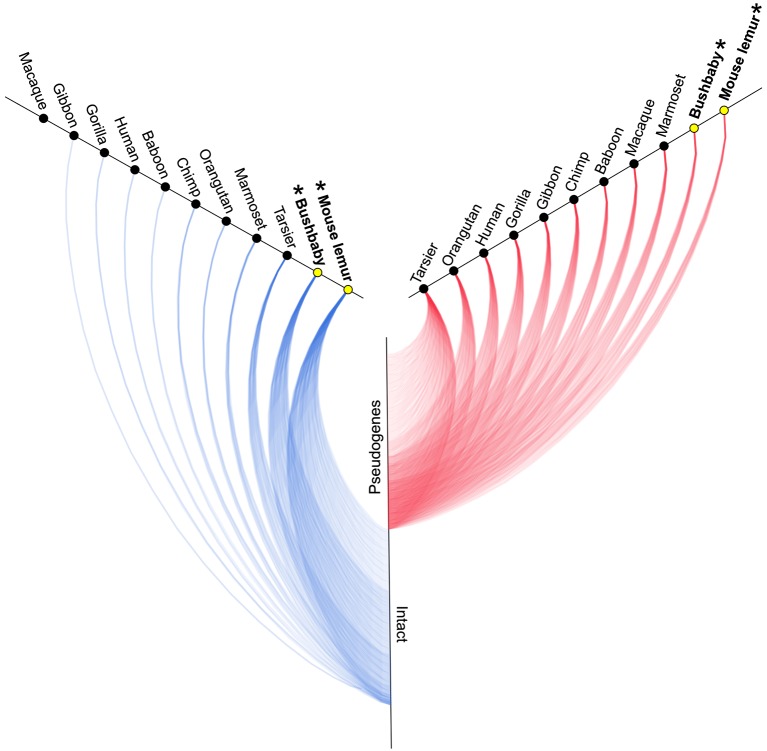
**Hive plot showing approximate number of intact (blue lines) and non-functional (red lines) V1R genes identified by Young et al. (2010) in primates (see Table 1)**. Asterisks and yellow circles identify strepsirrhine primates. See Table 1 for exact counts of gene copies.

A survey of the number of intact copies among the primates appears to substantiate this hypothesis (Figure 2; Table 1). Whereas the two strepsirrhines (mouse lemur and bushbaby) for which draft genomes exist show a preponderance of intact copies, the converse is true for the haplorrhine primates surveyed. In some cases, the lack of intact copies is complete, as in the macque, which shows zero intact copies (Table 1). Notably, some of the haplorrhine primates show a high number of copies overall, though the vast majority are pseudogenes (e.g., orangutan). This overall pattern is both phylogenetically and anatomically correlated. The strepsirrhine/haplorrhine divergence occurred at least 60 mya (Yoder and Yang, 2000, 2004; Poux and Douzery, 2004; Wilkinson et al., 2011; Springer et al., 2012), and is also reflected in the very different anatomies of the olfactory apparatus in the two primate groups. Indeed, the word “strepsirrhine” refers to the sinuous or “comma-shaped” nostrils of lemurs and lorises while “haplorrhine” refers to the simple or “single-fold” nostrils of tarsiers, monkeys and apes.

## The evolutionary footprints of ancestors and their descendants

An intricate mix of phylogenetic footprints and current adaptive landscapes have left their mark on primate V1Rs suggesting that the primate clade offers an ideal model system for exploring the molecular evolutionary and functional properties of the VNS of mammals. The primate phylogeny is neatly divided into halves, with the haplorrhines on one side and the strepsirrhines on the other. And whereas the haplorrhines show anatomical and behavioral characteristics that reflect their reliance on vision over olfaction, the opposite is true of the strepsirrhines. Of all extant primates, the strepsirrhine primates are renowned for their complex patterns of scent marking and other modes of olfactory communication (Schilling et al., 1990; Perret and Schilling, 1995; Perret, 1996, 2005; Kappeler, 1998; Perret et al., 2003; Suendermann et al., 2008; Boulet et al., 2010; Charpentier et al., 2010; Crawford et al., 2011; Delbarco-Trillo et al., 2011; Kappel et al., 2011; Rushmore et al., 2012). Moreover, all members of the Strepsirrhini retain the ancestral characteristic of a wet nose, typical of many mammals.

**Figure 3:**
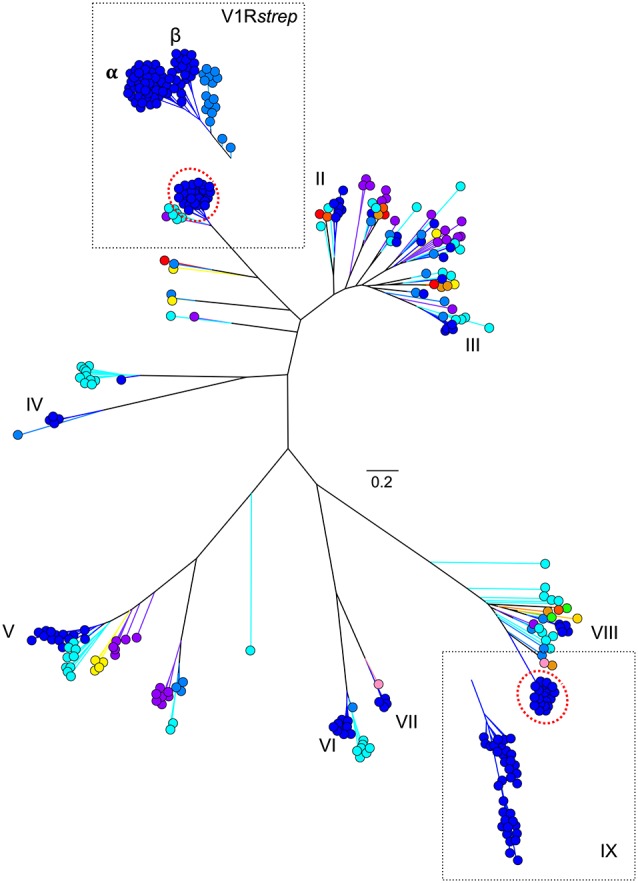
**Maximum likelihood tree (Figure 1B) with addition of novel V1R gene amino acid sequences identified from two V1R subfamilies by Yoder et al. (2014) and Larsen et al. (2014)**. Collectively, these analyses suggest the V1R*strep* and V1RIX repertoires within mouse lemur may be underestimated by approximately 25%. Refer to Figure 1A for color references.

As might be predicted, haplorrhines show a V1R system that is notably diminished both in terms of copy number and in terms of intact vs. pseudogene copies with the converse being true of the strepsirrhines (Figure 2; Table 1). Though this functional anatomical division has often been attributed to a shift from a nocturnal lifestyle in the ancestral primate to a diurnal one in the vast majority of haplorrhine primates (excepting only the living tarsiers and the monotypic owl monkey, genus *Aotus*), causality is unlikely to be that simple. Not only are the circadian habits of the ancestral primate currently debated, the intensive study of V1Rs in strepsirrhine primates has revealed a system in which history (i.e., phylogeny) appears to trump present-day lifestyle. Namely, diurnal strepsirrhines were found to have V1R systems that are equally elaborate to those of nocturnal strepsirrhines (Yoder et al., 2014). It is here worth noting, however, that our survey of primate V1Rs as illustrated in Figure 2 and Table 1 depend in large part on, and by necessity, on bioinformatic mining from draft genomes (Young et al., 2010). When more targeted sequencing approaches are implemented, we find that allele counts in draft genomes may be dramatically underestimated (Larsen et al., 2014; Figure 3). These findings are consistent with those from other mammalian groups. For example, Wynn et al. (2012) expanded the number of known mouse VR alleles by more than nine fold by employing massively parallel sequencing methods for VR genomic characterization. Even so, these authors noted that even though these methods were capable of accurately resolving more than half of mouse VRs, they also concluded that copy number variation and non-specific short read mapping compromise complete repertoire analysis. Clearly, much remains to be learned about the genomic characterization of VRs in primates and other mammals.

## Conclusions

In this review, we have synthesized information regarding gene family evolution across a broad phylogenetic expanse, spanning yeast to humans. Specifically, we have focused on the molecular-evolutionary dynamics of V1R genes in primates. As a result of these comparisons, we conclude that mammalian V1Rs are under extreme pressures for rapid evolution, both at the nucleotide and the amino acid levels. Moreover, these genes have been hypothesized to experience strong positive selection to maintain a diverse repertoire of functional copies when fine-scale and precise pheromonal communication is fundamental to species survival (e.g., predation) and/or propagation (e.g., mating behaviors). Alternatively, when other sensory systems such as vision are more dominant, positive selection is relaxed and the proportion of functional copies rapidly deteriorates, putatively due to the high rates of nonsynonymous substitution at the nucleotide level.

The distribution of intact to pseudogene copies in primates appears to bear out these assumptions. Whereas haplorrhine primates with their anatomically diminished olfactory systems show very few intact V1Rs, strepsirrhines with their ancestrally-retained and highly-developed olfactory anatomy show very large repertoires of intact V1Rs. Admittedly, limitations in available genomic resources for primates constrain our ability to draw definitive conclusions. In the near future, however, advancements in DNA sequencing technology (e.g., Pacific Biosciences *RS II*) will contribute to more robust primate genome assemblies (Chaisson et al., 2014) and, in turn, will provide more precise measures of gene family diversity, including V1Rs. In the meantime, we contend that the conservative nature of VR copy number estimates and the estimated ratios of intact to pseudogene copies yield patterns that will withstand further scrutiny with enhanced data. Primate V1Rs will likely persist in telling a story of ancestral function and divergent selection as species and clades have moved into ever diversifying adaptive regimes. The sensitivity to functional collapse in these genes, consequent to their precariously high rates of nonsynonymous substitution, confer a remarkable capacity to reveal the lifestyles of the genomes that they presently occupy as well as those of their ancestors.

## Conflict of interest statement

The authors declare that the research was conducted in the absence of any commercial or financial relationships that could be construed as a potential conflict of interest.
